# Nano-XRF of lung fibrotic tissue reveals unexplored Ca, Zn, S and Fe metabolism: a novel approach to chronic lung diseases

**DOI:** 10.1186/s12964-025-02076-4

**Published:** 2025-02-07

**Authors:** Bryan Falcones, Maik Kahnt, Ulf Johansson, Barbora Svobodová, Karin A. von Wachenfelt, Charlott Brunmark, Göran Dellgren, Linda Elowsson, Karina Thånell, Gunilla Westergren-Thorsson

**Affiliations:** 1 MAX IV Laboratory, Lund University, Lund, Sweden; 2 Lung Biology, Department of Experimental Medical Science, Lund University, Lund, Sweden; 3 Truly Labs AB, Medicon Village, Lund, Sweden; 4 Transplant Institute, Department of Cardiothoracic Surgery, Sahlgrenska University Hospital, Gothenburg, Sweden

**Keywords:** Synchrotron-radiation X-Ray fluorescence, Lung fibrosis, Elemental pathology, Metal metabolism, Iron trafficking

## Abstract

Synchrotron-radiation nano-X-Ray Fluorescence (XRF) is a cutting-edge technique offering high-resolution insights into the elemental composition of biological tissues, shedding light on metabolic processes and element localization within cellular structures. In the context of Idiopathic Pulmonary Fibrosis (IPF), a debilitating lung condition associated with respiratory complications and reduced life expectancy, nano-XRF presents a promising avenue for understanding the disease’s intricate pathology. Our developed workflow enables the assessment of elemental composition in both human and rodent fibrotic tissues, providing insights on the interplay between cellular compartments in chronic lung diseases. Our findings demonstrate trace element accumulations associated with anthracosis, a feature observed in IPF. Notably, Zn and Ca clusters approximately 750 nm in size were identified exclusively in IPF samples. While their specific role remains unclear, their presence may be associated with disease-specific processes. Additionally, we observed Fe and S signal colocalization in 650-nm structures within some IPF cells. Fe-S complexes in mitochondria are known to be associated with increased ROS production, suggesting a potential connection to the disease pathology. In contrast, a bleomycin-induced fibrosis rodent model exhibits a different elemental phenotype with low Fe and increased S, Zn, and Ca. Overall, our workflow highlights the effectiveness of synchrotron-based nano-XRF mapping in analyzing the spatial distribution of trace elements within diseased tissue, offering valuable insights into the elemental aspects of IPF and related chronic lung diseases.

**Supplementary Information:**

The online version contains supplementary material available at 10.1186/s12964-025-02076-4.

## Introduction

Idiopathic Pulmonary Fibrosis (IPF) is a chronic interstitial lung condition of unknown etiology, characterized by respiratory complications such as shortness of breath and dyspnea. It is partly caused by the fibrotic remodeling of the extracellular matrix (ECM) architecture and is associated with a very poor prognosis, with a median survival of around 3 years [1]. At the cellular level, IPF is thought to be initiated by harmful environmental triggers (such as the inhalation of toxic compounds, tobacco smoke, or viral exposure) in genetically predisposed epithelial and stromal cells in the distal lung [2]. The repetitive and progressive damage to the epithelium eventually triggers the recruitment of leukocytes such as macrophages and/or eosinophils, along with stromal cells like fibroblasts [3, 4]. The interaction of these cells in the distal parts of the lung, often through paracrine signaling, can lead to fibroblast differentiation into myofibroblasts, promoting tissue scarring of the interstitial lung in IPF.

The lungs are the first point of contact for atmospheric air and pollutants, which often include gases like ozone (O₃), nitrogen dioxide (NO₂), and carbon monoxide (CO). Pollutants trapped in lung tissue can undergo chemical changes, leading to the accumulation of elements such as metals, and causing conditions like anthracosis, characterized by black carbon deposits near the airways. Occupational exposure to these pollutants has been linked to an increased risk of chronic lung diseases like IPF [5]. Studies have identified elevated levels of metals such as iron (Fe), aluminium (Al), silicon (Si), manganese (Mn), and zinc (Zn) in polluted environments, contributing to respiratory diseases like COPD and lung cancer [6–7]. Despite these known associations, the exact contribution of these elements to the pathophysiology of chronic lung diseases like IPF remains unclear.

For example, it is known that Fe and Zn can be stored in organelles, and disruptions in their metabolism may trigger oxidative stress, which may contribute to fibrosis [8–9]. Further investigation is needed to explore the connection between elemental composition and IPF at the tissue level. X-ray fluorescence (XRF) mapping, particularly with advanced synchrotron sources, enables precise, high-resolution mapping of elemental concentrations in tissues. Nano-XRF, with its subcellular resolution, is a groundbreaking technique in biological research, including the study of mitochondrial disorders or neurological conditions such as Parkinson’s disease [10–11].

We hypothesize that the metabolism, trafficking, and accumulation of elements within endosomes and vesicles play a role in IPF. As a proof-of-concept for this notion and for future studies, we aimed to explore relevant chemical elements in human IPF lungs and in fibrotic rat lung samples using synchrotron-based nano-XRF mapping, alongside light and electron microscopy. Finally, to validate this methodology, we compared cryosectioning with room-temperature ultramicrotome sectioning. Our results highlight differences in the localization and accumulation of Zn, Fe, calcium (Ca), and sulfur (S) in IPF lungs compared to healthy samples.

## Results

### Preparation and XRF scanning workflow for IPF lung tissue

**Fig. 1:**
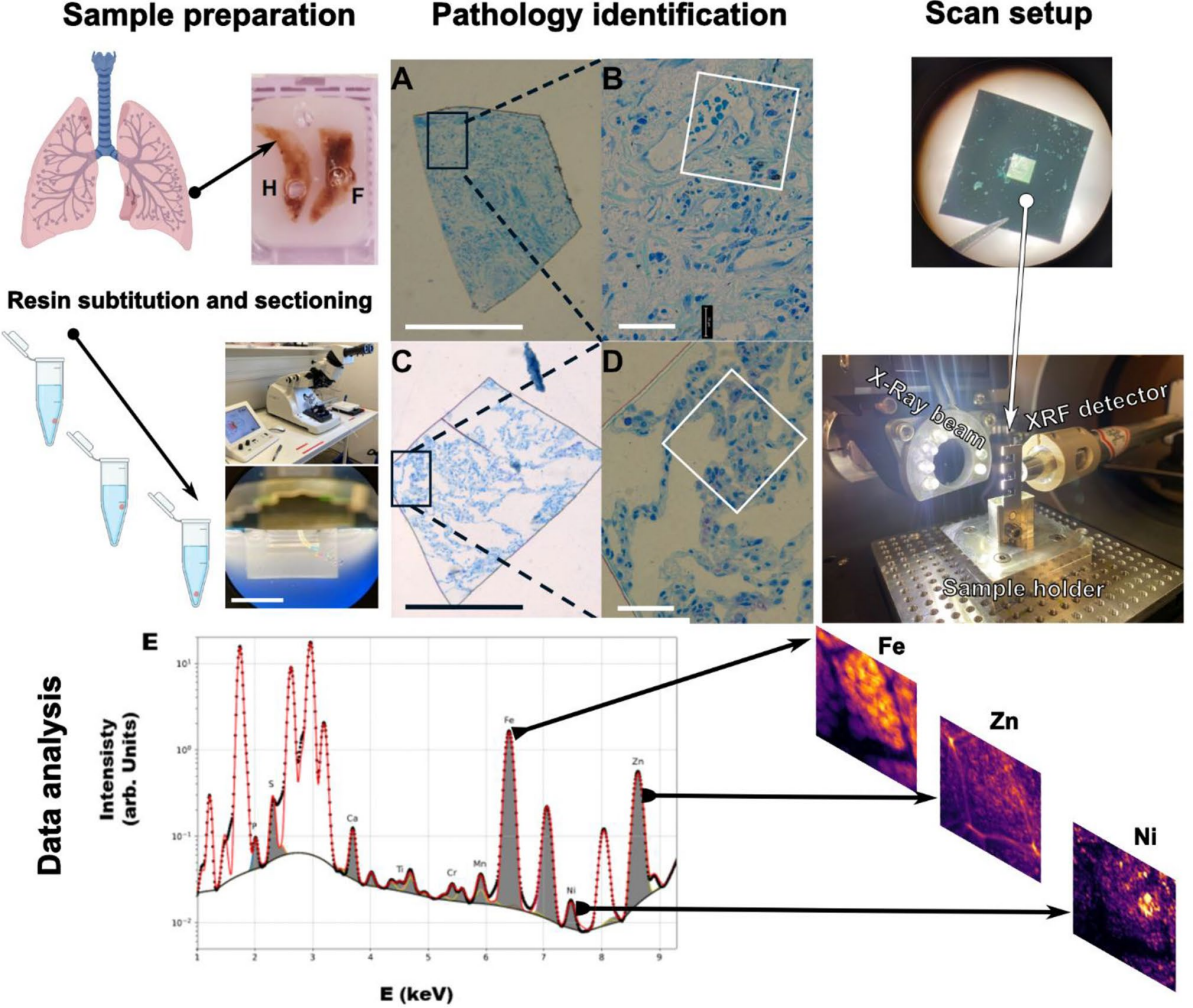
Workflow of the main steps to scan lung samples by nano-XRF at NanoMAX. **Sample preparation**: Illustrations depicting sample preparation: paraffin biopsy punch, resin exchange and close-up of the collection bath with a ribbon of samples (scale bar of the bottom picture 3 mm). **Pathology identification**: Microscopy images showing methylene blue staining for a healthy and IPF (**A**-**D**). Notice that B and D are the close-ups of the black rectangle. The white square shows an approximate area where the samples were scanned (scale bars, A and D 500 μm. B and E 50 μm). **Scan set up and data analysis**: Samples on 1 × 1 mm Si3N4 windows were scanned by a synchrotron X-Ray nanoprobe (100 nm) and later analyzed for the chemical elements present in the sample. **E**. Representative image of the XRF spectra of the scanned areas depicting the most relevant elements found. Further analysis allows the display of the elemental maps

Human lung tissue from healthy and IPF subjects were punched out from paraffin blocks, and the resin was substituted for semi-thin sectioning (Fig. 1, Sample preparation). 2-µm sections were stained with methylene blue to evaluate histology and select regions for scanning (Fig. 1, Identification of the pathology). These images revealed remodelled ECM-rich areas and reduced alveolar spaces characteristic of IPF, and were also used to navigate during scans.

1-µm sections were mounted on 1 mm² Si₃N₄ membranes (Fig. 1, Scan setup and data analysis) and scanned at NanoMAX (MAX IV Laboratory) using a synchrotron-based X-ray nanoprobe, which collected elemental fluorescence data per pixel. A representative sum XRF spectrum (Fig. 1E) shows the elements detected, with increased Zn and Fe peaks prompting further investigation.

**Fig. 2:**
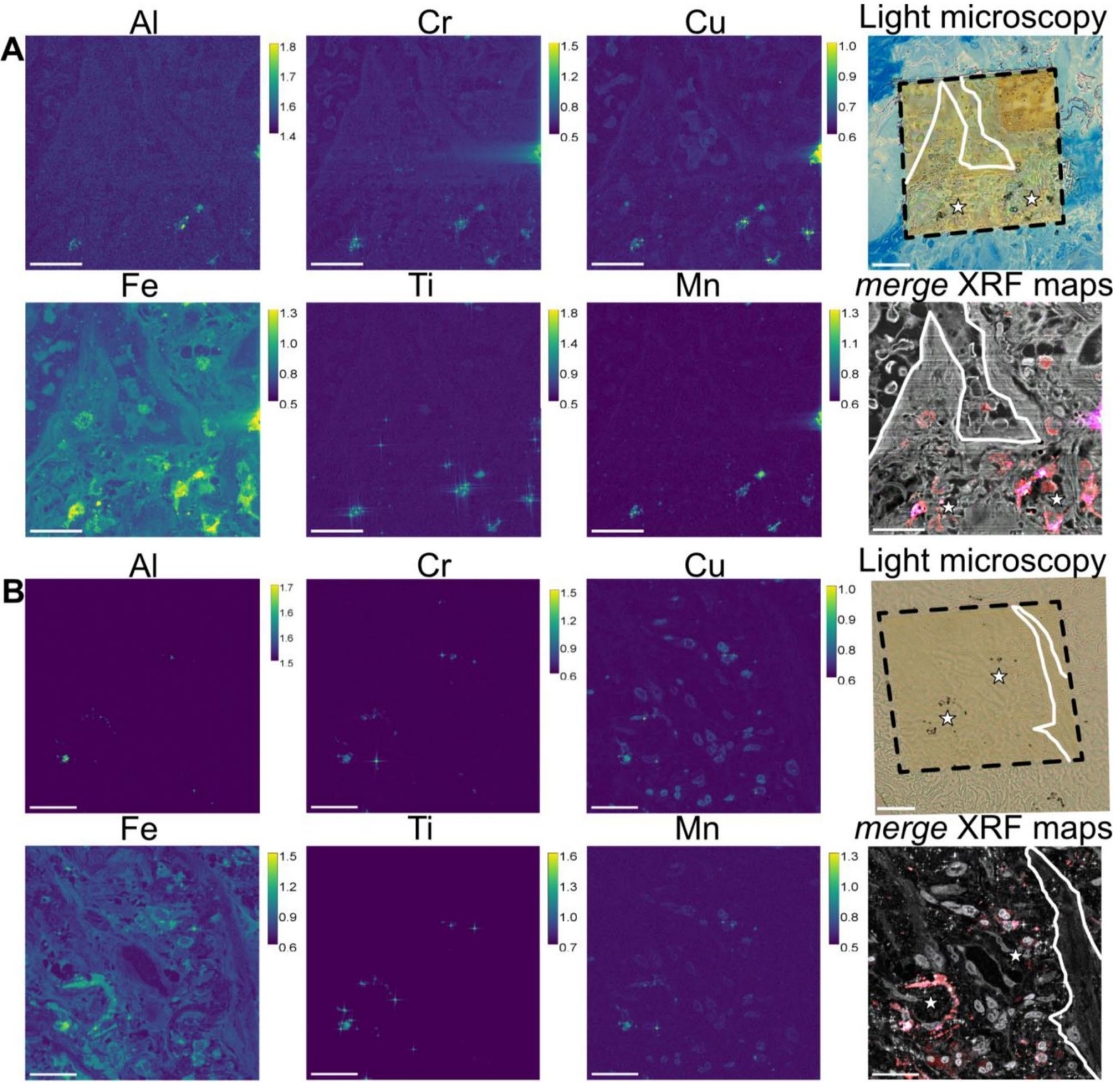
XRF maps of Al (aluminium), Cr (chromium), Fe (iron), Cu (copper), Ti (titanium), Mn (manganese). The calibration bar shows the ng/mm^2^ of the specific element. Light microscopy images showed exactly where the scans took place (black dotted squares) and white lines. They also highlight major structural features of the lung parenchyma (white lines). The merge XRF maps showed stack images from the individual XRF maps of Fe (red), Al (green), Mn (blue). Cl (chlorine, grey scale) shows a general view of the tissue. **A** and **B** depict different regions of the same patient. Scale bar is 20 μm. White star show the presence of anthracosis and metal accumulation

**Fig. 3:**
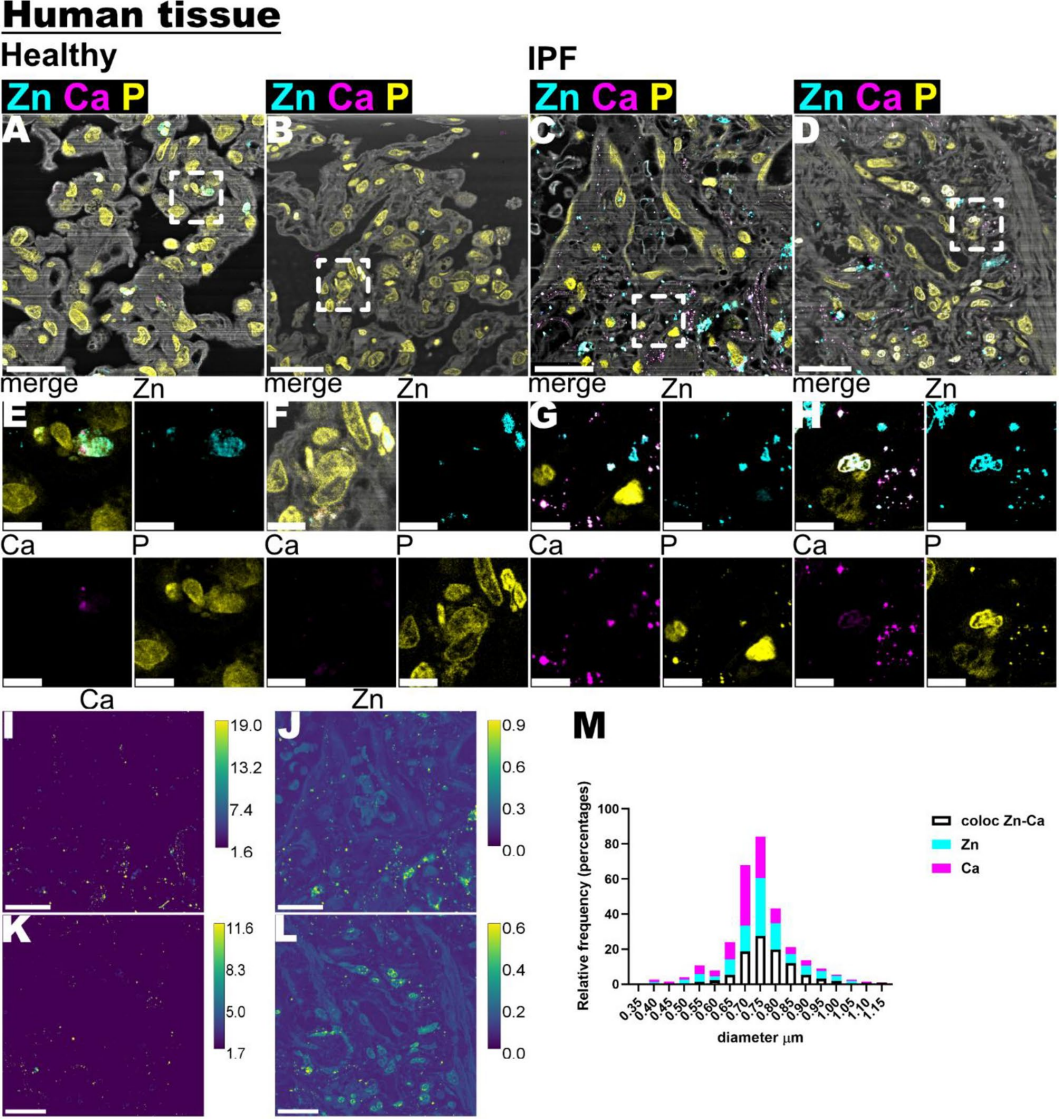
Zn (zinc) and Ca (calcium) image analysis of human IPF lung samples. **A**-**D** showed stacked images of Zn, Ca, P (phosphorous) and Cl (chlorine) XRF maps. Scale bar is 20 μm. **E**-**H**. Showed close-up views of Zn and Ca XRF maps from images A-D (white dotted squares) Scale bar is 5 μm. **I**-**L**. XRF maps of Zn and Ca of lung IPF, the calibration bar display ng/mm^2^. Scale bar is 20 μm. M. Histogram distribution illustrating the size frequency of high-concentrated Zn and Ca clusters

**Fig. 4:**
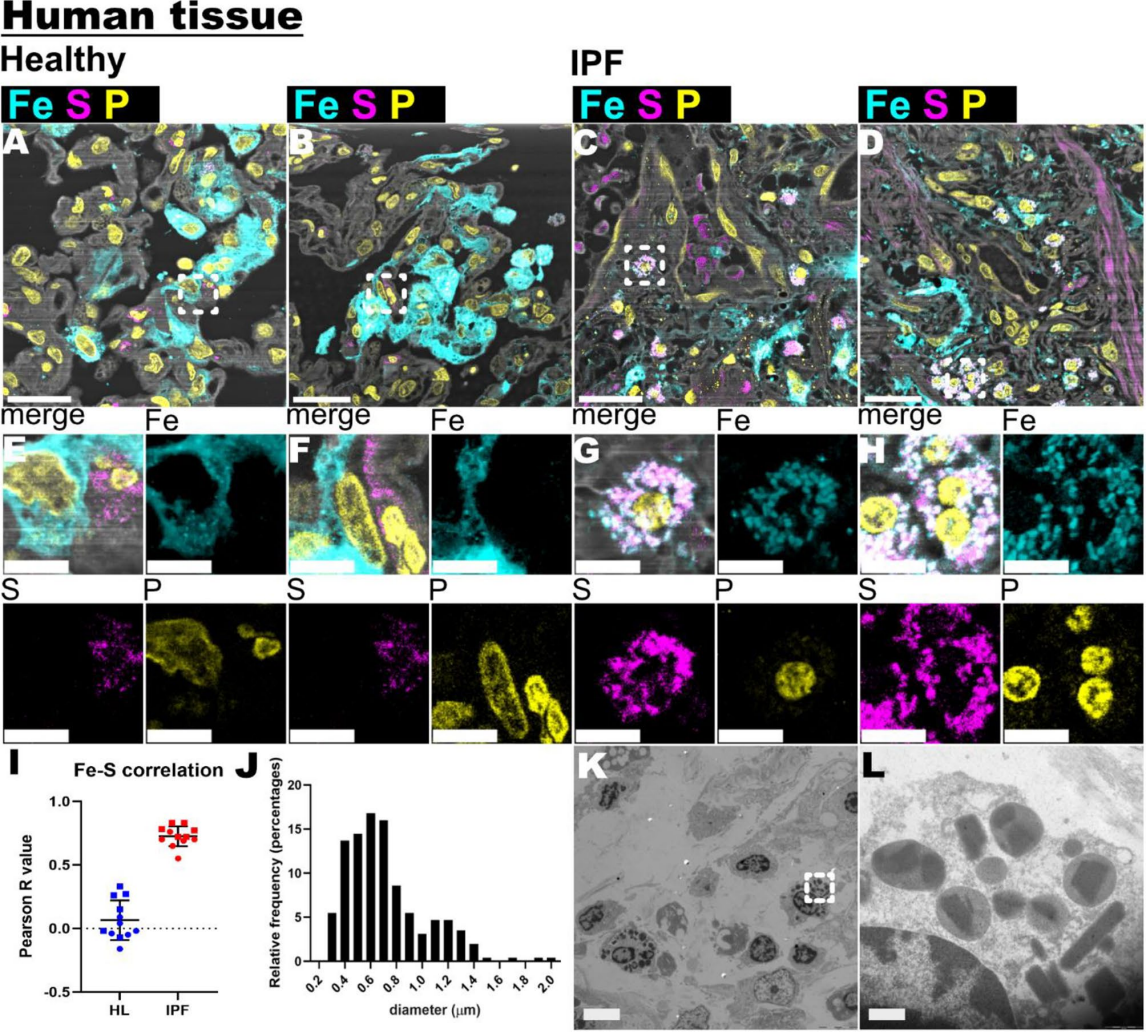
Fe (iron) and S (sulfur) image analysis of IPF human lung samples. **A**-**D** showed stacked XRF maps of Fe, S, P (phosphorous) and Cl (chlorine) XRF maps. Scale bar 20 μm. **E**-**H** shows close-up views of Zn and Ca XRF maps from images A-D (white dotted squares). Scale bar is 5 μm. **I**. Fe-S co-localization analysis. Each data point correspond to an individual cell. 12 Healthy cells and 12 IPF cells were analyzed. **J**. Histogram distribution illustrating the size frequency of Fe-S co-localized structures. **K**. TEM image of adjacent IPF lung sections to those measured by nano-XRF. Scale bar is 5 μm. **L**. TEM close-up view of the Fig. 3K (white dotted square). Scale bar is 500 nm

### Anthracosis of the IPF lung parenchyma is heterogeneously coated with trace metals

The scans revealed the presence of more than 15 elements distributed within the samples (refer to Supplementary Files, Fig. S2 for all XRF maps). An overview article on XRF maps of relevant trace elements in health and disease [12] highlights spatial localizations that are similar to our findings in IPF samples. Multi-metal XRF maps of IPF scans, shown in Fig. 2A-B, indicate that the highest intensity points are concentrated in confined surface areas of the IPF parenchyma (white stars Fig. 2A-B). Light microscopy images of the scans revealed the presence of anthracosis, visible as black particles within the regions where the scans were performed. In the merged XRF maps, we confirmed that the spatial distribution of the detected metals corresponds to the anthracosis. Notice that “star-like” patterns appear in some of the XRF maps where the signal is more intense (Fig. 2). This phenomenon occurs due to the beam shape which is not a single round spot but includes weak tails extending from its bright centre, which are visible in log scale. When scanning over a small particle with high elemental content, the weak tails interact with the particle border as the beam centre raster the sample, producing detectable XRF photons and creating the “star-like” pattern in log-scaled maps.

### Zinc and calcium localization display high-concentrated clusters in the IPF parenchyma

Figures 3A-D display stacked XRF maps of phosphorus (P), chlorine (Cl), calcium (Ca), and zinc (Zn). First, we observed Ca hot-spots in the healthy tissue samples, while Zn appeared more prominent, particularly within the nuclei (identified by high-intensity phosphorus signals) in both healthy and IPF samples (Fig. 3E-H)

The most notable difference in Zn and Ca was the accumulation in the form of hot spots in the IPF tissue samples, compared to the healthy samples. XRF maps of Zn and Ca concentrations in IPF (Fig. 3I-L) revealed that these clusters exhibited high values in the range of ng/mm² and occasionally co-localized. Segmented hot spots (individual and co-localized) were analysed for size, with a majority found to be approximately 750 nm, as shown in the histogram distribution in Fig. 3M.

### Iron and sulfur co-localization reveals a novel cell phenotype

The XRF maps of iron (Fe) and sulfur (S) showed distinct localization patterns within healthy and IPF samples (Fig. 4A-D). Interestingly, certain cells in IPF lungs exhibited a high concentration of S, which seemed to co-localize with Fe, as evidenced by close-up views of the individual XRF maps (Fig. 4E-H). Fe observed near the cell nuclei appeared to be confined within defined compartments, reminiscent of intracellular cytoplasmic structures. Individual cells who were S positive were analysed for pixel-based co-localization of Fe-S, revealing an increased Pearson correlation coefficient in IPF compared to healthy samples (0.72 ± 0.15 vs. 0.06 ± 0.07) (Fig. 4I-J). Subsequent analysis of the size distribution of Fe-S + structures revealed dimensions ranging from 0.4 to 0.7 μm, constituting 60% of the total number of detected structures. Furthermore, TEM images revealed high-contrast granules that contained an angular, denser feature, reminiscent of a crystalloid. Interestingly, the sizes of these granules (Fig. 4K-L) were similar to the Fe-S + structures.

**Fig. 5:**
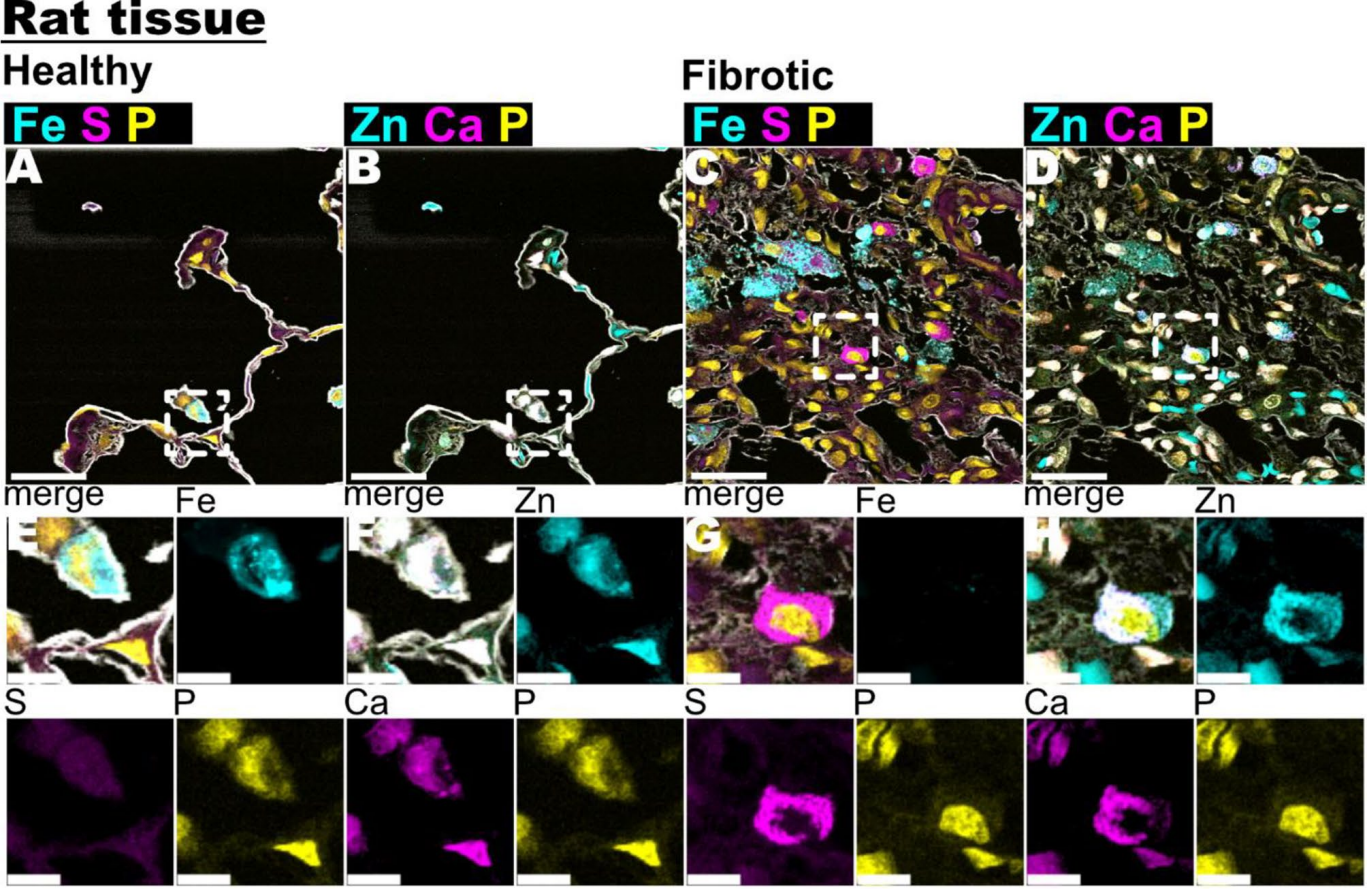
Fe, S, Zn and Ca image analysis in fibrotic rat lung tissue. **A**-**D** showed stacked XRF maps of Fe, S, P (phosphorous) and Cl (chlorine) XRF maps, respectively. Scale bar is 20 μm. **E**-**H** shows close-up views of Zn and Ca XRF maps from images **A**-**D** (white dotted squares). Scale bar is 5 μm

To explore the potential nature of Fe-S clusters in IPF, fibrotic lung tissue from a bleomycin-induced rat model, as well as a pro-fibrotic but otherwise healthy area of the same animal, were scanned, and Fe, S, Zn, and Ca XRF maps were displayed (Fig. 5A-E). In Fig. 5C and G, certain cells within the fibrotic lung parenchyma showed elevated levels of sulfur. However, unlike the human samples (Fig. 4G-I), no co-localization of Fe-S was observed (Fig. 5A&G). Interestingly, S + cells in the fibrotic XRF maps of Ca and Zn (Fig. 4D&H) exhibited increased intracellular levels rather than appearing as individual clusters as seen in Fig. 2.

### Ultrasectioning vs. cryosectioning comparison

**Fig. 6:**
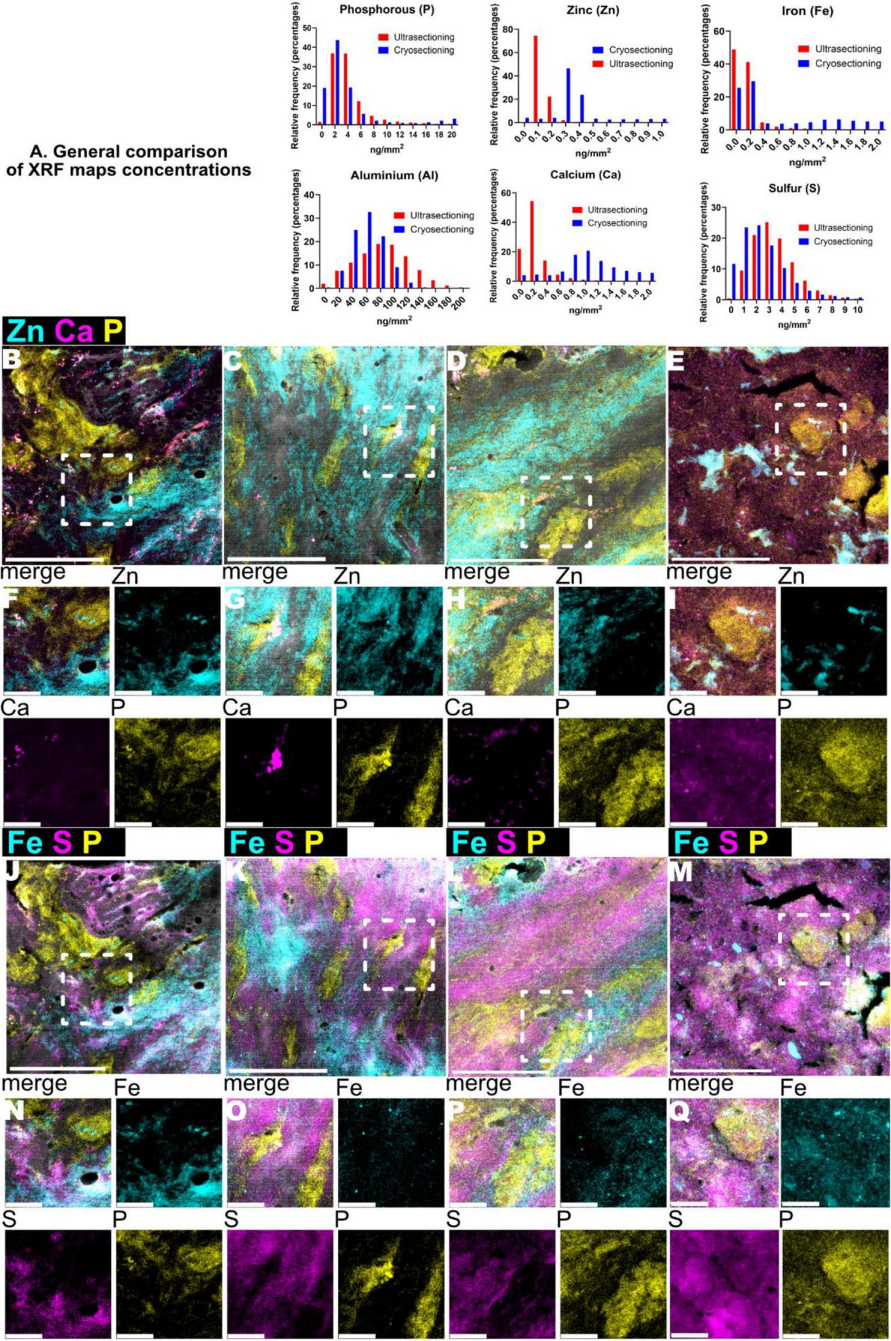
Comparison of Ultrasectioning and cryosectioning sample preparation methods. **A**. Histograms of the concentration (in percentages) for each pixel for the most relevant elements of this study: phosphorous (P), zinc (Zn), iron (Fe), sulphur (S), calcium (Ca) and aluminium (Al). **B**-**E** showed stacked images of Zn, Ca, P (phosphorous) and Cl (chlorine) XRF maps of cryosectioned samples. Scale bar is 20 μm. **F**-**I**. Showed close-up views of Zn and Ca XRF maps from images **A**-**D** (white dotted squares) Scale bar is 5 μm. **J**-**M** showed stacked XRF maps of Fe, S, P (phosphorous) and Cl (chlorine) XRF maps. Scale bar 20 μm. **E**-**H** shows close-up views of Zn and Ca XRF maps from images **A**-**D** (white dotted squares). Scale bar is 5 μm

To validate our sample preparation, Fig. 6A compares two methods of sample sectioning, specifically, cryosectioned samples (Cryosectioning) and chemically-fixated ultrasectioned at room temperature (Ultrasectioning), highlighting similarities and differences for some of the elements detected. P and S show similar frequency profiles, while zinc and calcium exhibit notable shifts, with higher concentrations in cryosectioning. Fe and Al display comparable concentrations but differ in frequency profiles. Stacked maps reveal additional distinctions; P maps from cryosectioning (Fig. 6B-I) show less sharp and enlarged features (e.g., nuclei) compared to ultrasectioning. Zn and Ca maps differ, with ultrasectioning (Fig. 6C-D, G-H) showing distinct hotspots, while cryosectioning reveals clustered Ca but more uniform Zn distributions. Cryosectioning Fe maps (Fig. 6J-Q) display some accumulation, unlike S, and no co-localization of these elements is observed, consistent with Fig. 3A-H.

## Discussion

In this study, we showcase the use synchrotron-based nano-XRF mapping to study the elemental distribution in idiopathic pulmonary fibrosis (IPF) lung tissue compared to healthy samples and show specific changes in the distribution of calcium (Ca), zinc (Zn), iron (Fe), and sulfur (S). Using radiation-based nano XRF mapping, we identified differences in elemental clustering and co-localization patterns, which may provide new insights into the pathophysiology of IPF.

Our findings revealed Zn and Ca accumulation and formation of clusters in IPF tissue, which were significantly larger and more frequent than those observed in healthy lung samples. Disturbances in metal homeostasis have previously been associated with oxidative stress and tissue damage [13, 14]. The co-localization of Zn and Ca observed in this study highlights a distinctive elemental distribution in fibrotic tissue, providing a basis for future studies to explore their significance.

In addition to Zn and Ca, we observed a strong correlation between Fe and S in IPF samples, with a Pearson correlation coefficient higher than in healthy tissue. The Fe-S clusters in IPF tissues were similar in size to high-contrast granules seen in TEM images. These granules contained dense structures reminiscent of lamellated crystalloids, which are highly characteristic of eosinophils but can occasionally be found in lysosome-related structures of macrophages [15] These findings align with previous research linking Fe to oxidative stress, inflammation, cell death (ferroptosis), and fibrosis [16, 17]. However, the exact nature and role of these Fe-S clusters in the disease process remain to be elucidated.

When we applied the same XRF mapping techniques to a bleomycin-induced rat model of lung fibrosis, we observed differences in Fe-S co-localization compared to human IPF samples. This could indicate that the rat model replicates certain aspects of lung fibrosis, while it does not fully mirror the elemental distribution seen in human disease. Furthermore, the increased intracellular levels of Zn and Ca in the rat model differed from the clustering patterns in human IPF tissue, highlighting potential species-specific variations in fibrosis progression.

Limitations of this study include sample preparation, where element loss may occur due to fixation, dehydration, and embedding. In regards of this topic, we compared scans from cryosectioned and ultrasectioned in Fig. 6. Although ultrasectioned samples show attenuated concentration values, the differences between methods do not indicate significant elemental leaching during preparation. Despite using identical scanning parameters at NanoMAX, differences in the maps suggest an influence of sample thickness. Nano-XRF focus depth depends on factors like fluorescence energy and sample thickness, with 10 μm cryosections potentially affecting resolution and accuracy. Thicker samples can absorb or scatter X-rays, reducing signal detection from deeper layers and increasing interaction volume, which blurs depth resolution and averages signals [18]. Optimizing cryosection´s thickness could improve nano-XRF sample preparation. Another limitation is the limited accessibility to synchrotron facilities, reducing the number of scans and biological variability assessed. Future studies using larger cohorts and correlative spatial imaging techniques, such as Laser Ablation Inductively Coupled Plasma Mass Spectrometry (LA-ICP-MS) and spatial proteomics (e.g., multiplex immunofluorescence), will be crucial for further elemental and molecular characterization in IPF.

In conclusion, our study shows elemental changes involving Zn, Ca, Fe, and S in IPF tissue. These findings showcase the utility of synchrotron-based nano-XRF mapping for examining the spatial distribution of trace elements in disease tissue samples. Nevertheless, it also opens avenues for the integration of X-Ray Imaging into the field of respiratory disease research, and we see it as a foundational step toward linking the molecular and elemental information in tissue.

### Experimental section

#### Patient material

Lung explants from one non-smoking healthy donor and one formerly smoking IPF patient, both 68-year-old males, were obtained from Sahlgrenska University Hospital in Gothenburg. The study was approved by the Swedish Research Ethical Committee in Gothenburg and all experimental protocols were carried out in accordance with guidelines approved by the ethical committees.

#### Bleomycin-induced fibrotic rat model

Sprague–Dawley male rats (7–10 weeks of age (250–400 g in weight) from Taconic (Taconic, Lille Skensved, Denmark) were kept at 12 h light/dark cycles and fed *ad libitum*. All animal studies were ethically reviewed and carried out in accordance with European Directive 2010/63/EEC and ARRIVE guidelines [19] and experimental procedures were evaluated and approved by the local ethical committee in Lund/Malmö, Sweden, with permit number 3226/2017. The rats received a single intra-tracheal dose of 1000 U bleomycin (Sigma–Aldrich, St. Louis, MO, United States) in 200 µl saline. After an initial 7–10 days of inflammation the rats developed progressive fibrotic disease.

#### Sample preparation for XRF

Samples from human lung explants (one **healthy** and one **IPF**) and rat lungs (one bleomycin-treated) were fixed in paraformaldehyde (4%), dehydrated in increasing ethanol dilutions, embedded in paraffin blocks and reserved until use. 3 mm diameter samples were punched out of the human and of the rat paraffin tissue blocks (regarding the rat tissue block, the fibrotic and the healthy biopsies were punched out from different areas the same bleomycin-treated animal depicted by hematoxylin and eosin staining evaluation). The tissue was then deparaffinated, dehydrated in acetone and re-embedded in Poly/Bed 812 Embedding Media (Polyscience Europe GmbH, Eppelheim, Germany) (Fig. 1, Sample preparation). 2-um thick slices were sectioned and initially evaluated by methylene blue staining (Fig. 1). Afterwards two different 1-µm thick sections from different distal regions per sample were placed on 1000-nm thick Si_3_N_4_ windows (Norcada, Canada).

#### Nano-XRF scanning

XRF scans from two healthy and two IPF human samples as well as one healthy and one fibrotic rat sample, were carried out at the diffraction end station [20] of the hard X-ray nanoprobe beamline NanoMAX [21] at the MAX IV Laboratory (Lund, Sweden). An incident photon energy of 10 keV was chosen to excite elements from Al up to Zn in the periodic table with a 90 nm^2^ beam spot size and 1.5 × 10^11^ photons/s of flux. At each exposure point, XRF photons emitted by the sample were recorded by a Sirius-SD one-element detector (RaySpec, UK) positioned under 90 degrees to the incoming beam (in the direction of its linear polarization) to minimize the measurement background, with the sample angled 15^o^ away from normal incidence. The pulses from the silicon drift detector were analyzed by a high-performance pulse processor (Xspress3, Quantum Detectors, UK) (Fig. S1). Up to 100 µm^2^ sized areas of each sample were recorded at 100 nm step size (and 200 nm for rat samples) and 100 ms exposure time per point. This accumulated to about 24 h of measurement time per map. The beam intensity incident on the sample was simultaneously recorded with the XRF signal and all data points were normalized to the beam intensity. All measurements were conducted in air and at room temperature. Additional information of the workflow and a scheme of the beamline setup can be found in Fig. 1, Scan setup.

#### XRF quantification and image analysis

Concentration maps for all relevant elements were calculated by fitting of the sum spectra (Fig. 1E) using the PyMCA software [22] (version 5.6.3) and an XRF reference sample (RF-C00-X, AXO-Dresden, DE), which was measured with the same experimental parameters. The resulting maps of the measured sample area show element concentrations for each measured position expressed in ng/mm^2^. All further analysis was performed on these maps using the image analysis software Fiji (v.2.14) [23]. Figure 1 maps are represented in a pseudo log-scale for better assessment of the features in the image. The Zn, Ca, Fe and S segmentation and quantification of Figs. 2, 3 and 4 workflow can be found in the Supplementary methods.

#### Cryosectioning vs. ultrasectioning

Distal explants from IPF donors were snap-frozen in liquid nitrogen, embedded in OCT, and cryosectioned at 10 μm thickness using a cryostat with the knife and object maintained at -15 °C and − 17 °C, respectively. Sections were mounted on 1000-nm Si₃N₄ windows at room temperature. Four scans were performed with a 45 × 45 μm field of view, using the same settings as described previously. Absolute concentration values were extrapolated for comparison with ultrasectioned samples.

XRF maps of the most relevant elements in this study—iron, sulfur, zinc, calcium, aluminum, and phosphorus—were generated from cryosections (Cryosectioning). The frequency of element concentrations across all pixels was compared to XRF maps obtained from ultrasectioned blocks of the same donor (Ultrasectioning). Additionally, XRF maps of zinc, calcium, and phosphorus, as well as iron, sulfur, and phosphorus, were processed as Figs. 2A-H and 3A-H, stacked into 2D files and provided a close-up view of the images.

#### Data and statistics

Data from Fe-S correlation was expressed as mean ± SD of Pearson coefficient per cell, respectively. The detected clusters of Zn, Ca and Fe were depicted as histogram of their abundance in percentages, respectively. No statistical analysis was used. All graphs were prepared using GraphPad Prism Software (San Diego, California, USA).

## Electronic supplementary material

Below is the link to the electronic supplementary material.

Supplementary Material 1

## Data Availability

No datasets were generated or analysed during the current study.
