# Supplementary material for: Nano-XRF of lung fibrotic tissue reveals unexplored Ca, Zn, S and Fe metabolism: a novel approach to chronic lung diseases
Source: Cell Commun Signal. 2025 Feb 7;23:67. doi: 10.1186/s12964-025-02076-4 (PMC11806689; doi:10.1186/s12964-025-02076-4)
Supplement: Supplementary file 1 — Supplementary Material 1 [file 12964_2025_2076_MOESM1_ESM.docx]

Nano-XRF of Lung Fibrotic Tissue Reveals Unexplored Ca, Zn, S and Fe Metabolism: A Novel Approach to Chronic Lung Diseases.

Bryan Falcones^1,2,#^, Maik Kahnt^1^, Ulf Johansson^1^, Barbora Michaliková^2^, Karin A von Wachenfelt^3^, Charlott Brunmark^3^, Göran Dellgren^4^, Linda Elowsson^2^, Karina Thånell^1^*, Gunilla Westergren-Thorsson^2^*.

*1. MAX IV Laboratory, Lund University, Lund, Sweden
2. Lung Biology, Dept. of Experimental Medical Sciences, Lund University, Lund, Sweden
3. Truly Labs AB, Medicon Village, Lund Sweden
4. Transplant Institute and Department of Cardiothoracic Surgery, Sahlgrenska University Hospital, Gothenburg, Sweden
*Contributed equally ^#^Corresponding author*

**Supplementary methods**

**Bleomycin-induced fibrotic mouse model.**

Upon arrival, the rats underwent a health check and were allowed to acclimatize to the housing conditions before receiving a single intra-tracheal dose of 1000 U bleomycin (Sigma–Aldrich, St. Louis, MO, United States) in 200 µl saline. The intra-tracheal administration was performed by placing the lightly anesthetized (IsoFloQR vet, Orion Pharma, Sweden) rat, in a supine position on a slanting board using a syringe connected to a blunt cannula with a small steel marble at the top. At termination, the rats received an intraperitoneal overdose of Pentobarbital Sodium (Apotek Produktion and Laboratorier AB, Sweden). Subsequently, BAL was performed by flushing the lungs with PBS at a pressure of 10 cm H_2_O for 2 min and collecting the lavage fluid. This procedure was repeated two times and the left lung was ligated and insufflated with 4% paraformaldehyde and subsequently paraffin-embedded.

**Trace metal analysis**

To study the presence of trace elements in IPF as seen in Fig. 1, IPF XRF maps of Titanium (Ti), chromium (Cr), iron (Fe), aluminium (Al), manganese (Mn) and chlorine (Cl) were displayed in a pseudo log scale by first transforming to a logarithmic scale, multiplied by 0.1 and added 1. The purpose of the mathematical transformations is the display of positive values in the calibration bar (Figure 1). We noticed that chlorine (Cl) showed the architecture of the lung parenchyma, and therefore chose to combine it with other maps in further analysis.

**Zn and Ca hot-spots accumulation**

The individual XRF maps of Zn and Ca for healthy and IPF were plotted in a multicolour z-stacked image (Fig. 2). The individual maps of Zn and Ca were changed to 8-bit of depth (to allow the use of the following plugin) and segmented by using the same threshold “Minimum”. The resultant images were merged into a single image and analysed using the ComDet v.0.5.5 plugin for ImageJ (https://github.com/UU-cellbiology/ComDet) to quantify the amount of individual clusters and whether they were localizing. The settings of the plugins used were done consistently to all the images for unbiased comparison. The area of the detected clusters were transformed (µm^2^) into diameter of the cluster (assuming a sphericity of near 1) and plotted their frequency in histograms (Fig. 3 E).

**Cell-specific analysis of Fe and Sulphur colocalization**

Fe, sulphur (S) and phosphorous (P) maps were stacked in a single file to illustrate the distribution within the lungs. We noticed an increased accumulation of S within some IPF cells which were stored as ROIs and generated individual masks of the cells, 6 replicates per scan. The same amount of high-concentrated cells were also selected as ROIs in the healthy scans as controls. XRF tiff files of Fe and S were changed to 8-bit of depth and segmented by applying a threshold. The resultant images within the selected masks were analysed for co-localization by a plugin in Fiji for a pixel intensity-based co-localization “coloc 2” (https://github.com/fiji/Colocalisation_Analysis) which is based on a previous publication elsewhere [1]. The plugin allows the automatic generation of the scatter plots and the images of highlighted co-localized pixels and the (Fig. 4 I-J). The segmented Fe images were applied the watershed tool and analyse the segmented granules of Fe.

**Supplementary results**

**Figure S2**. XRF maps from run 1 &2


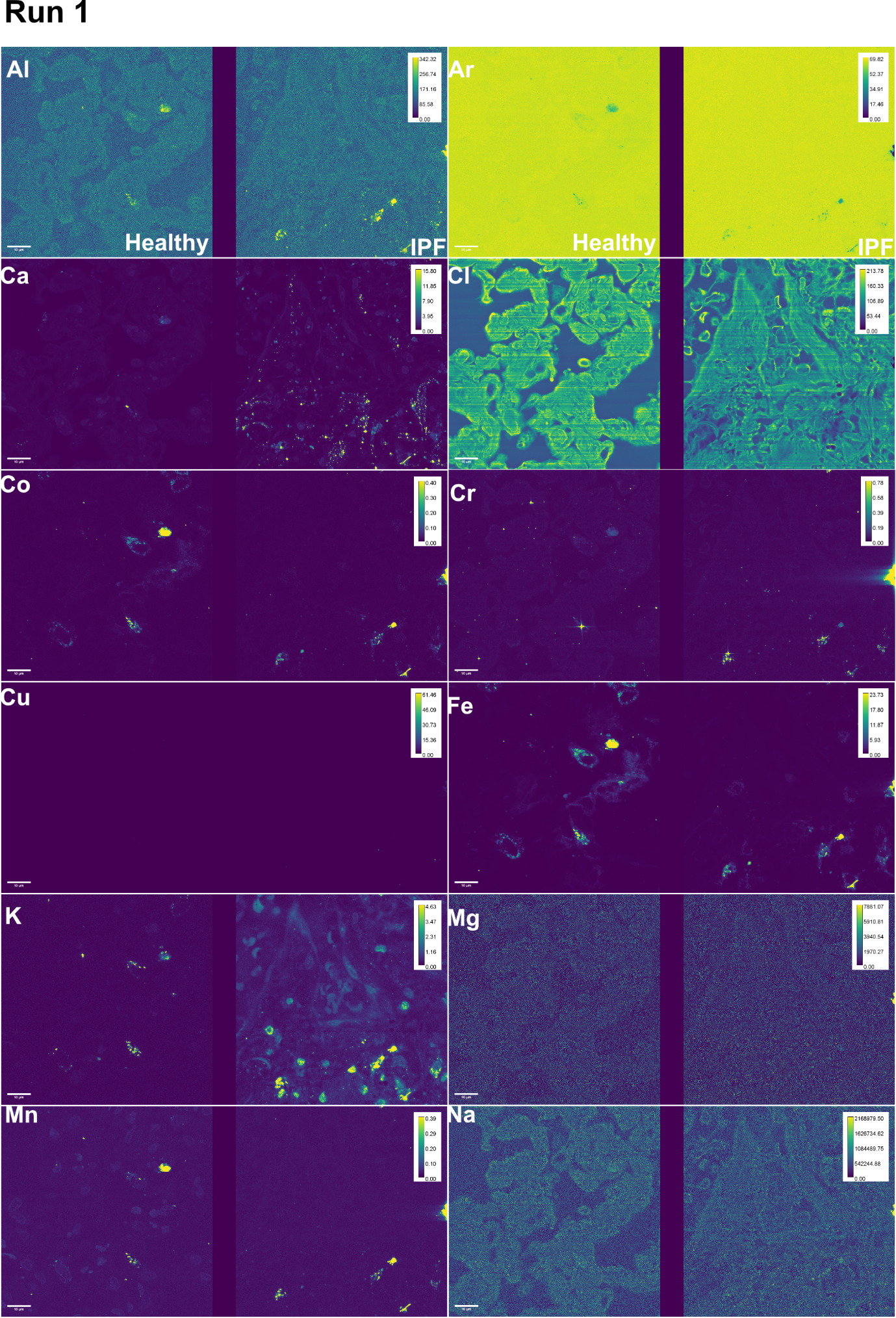


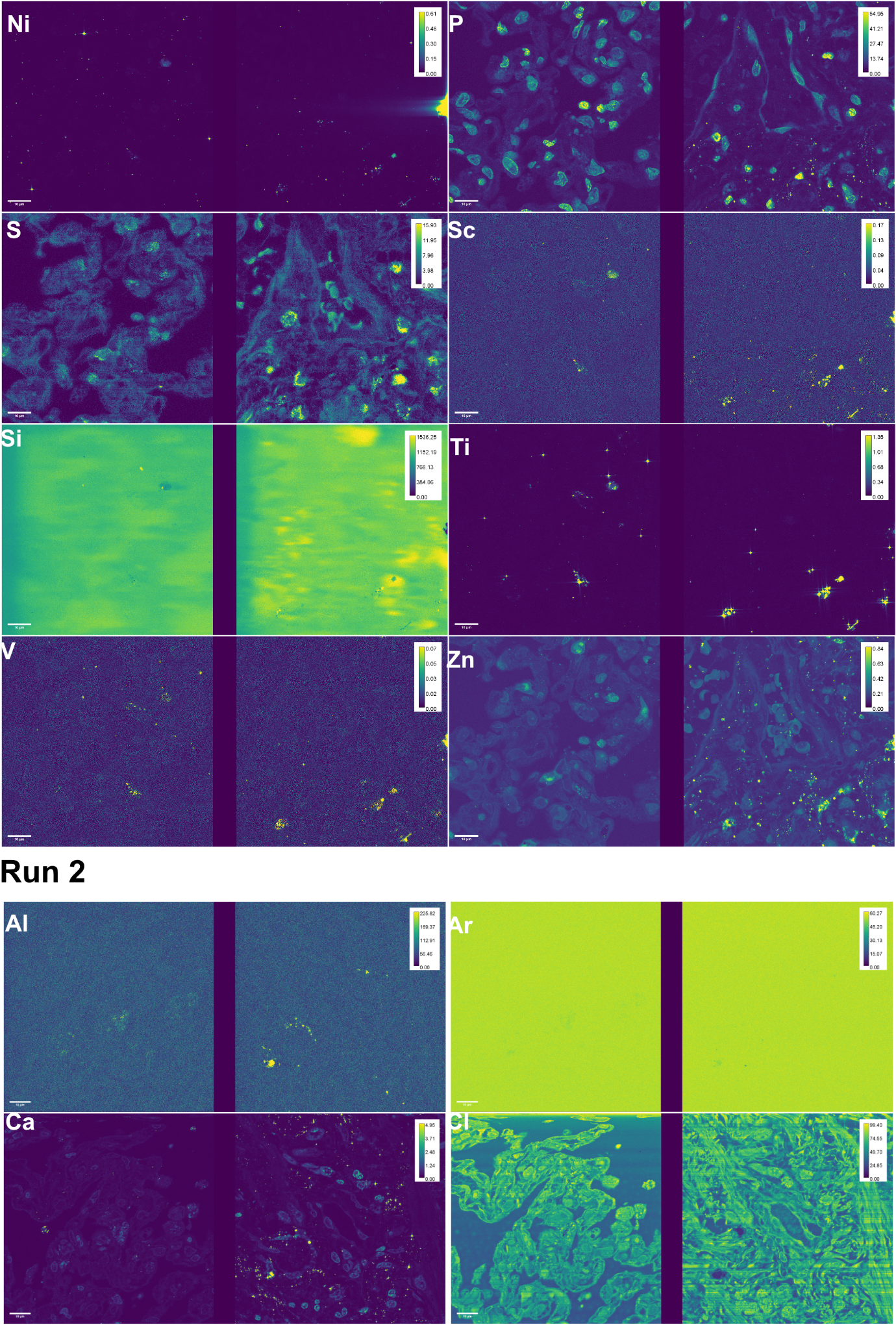


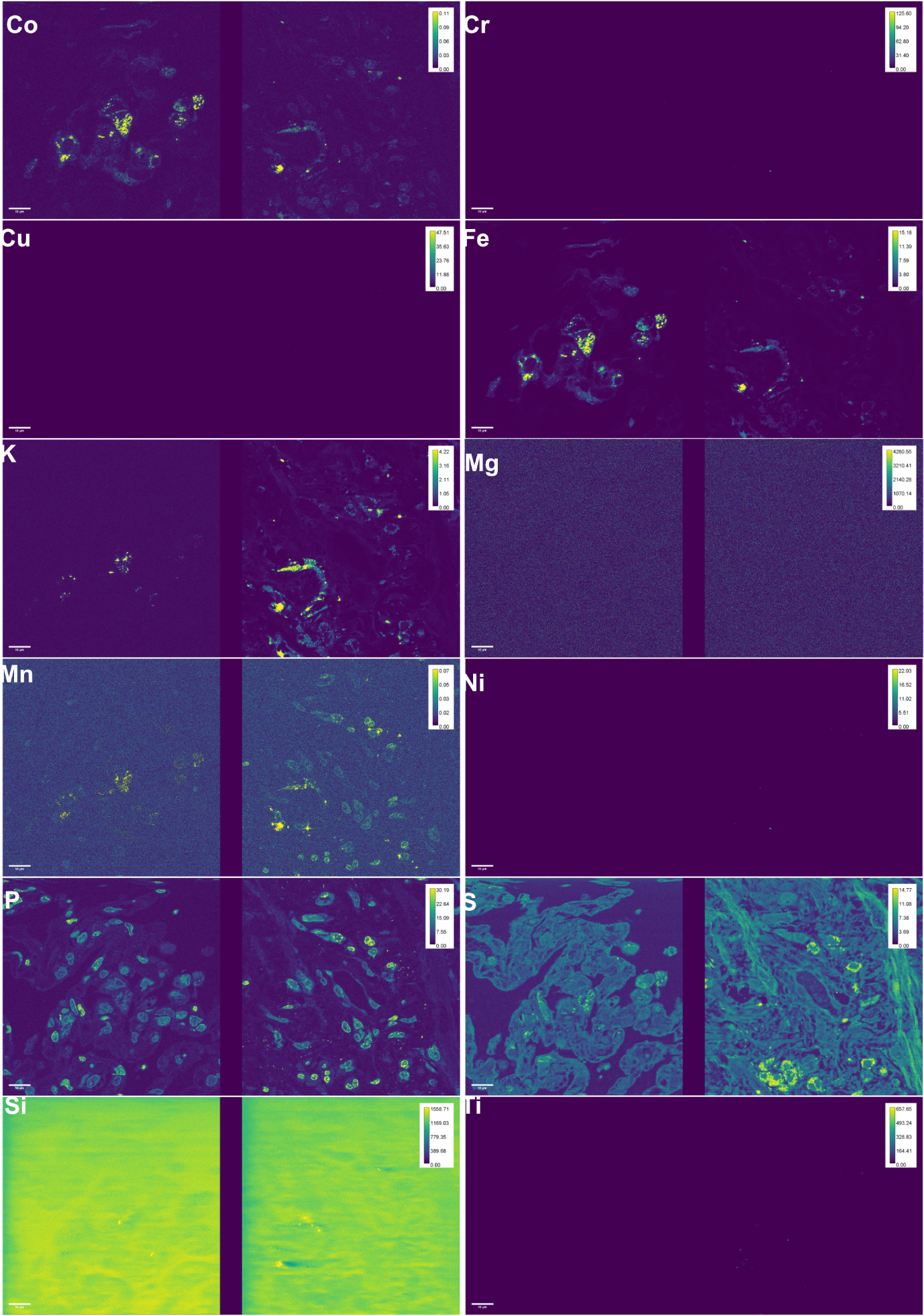


Zn


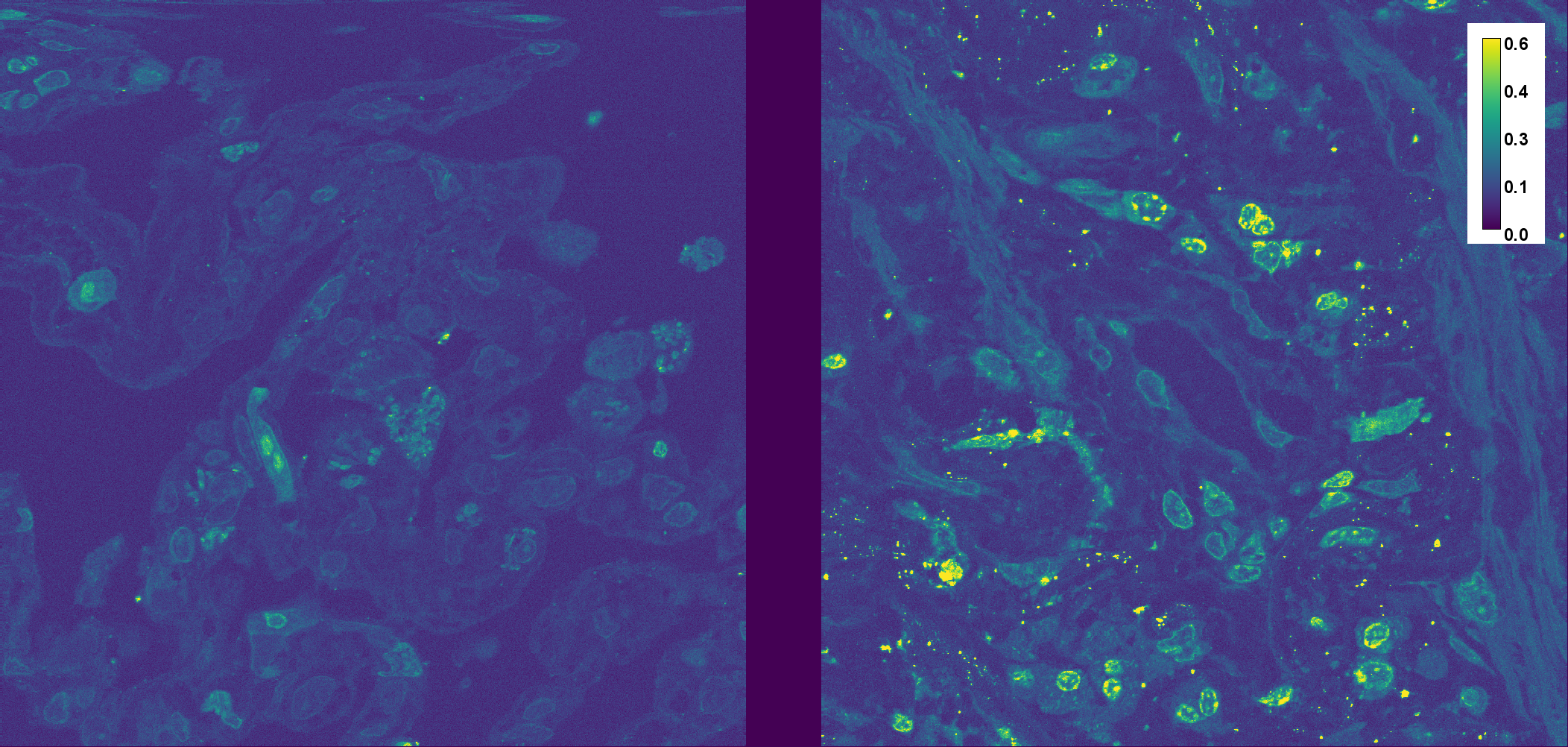


**References**

[1] S. V. Costes, D. Daelemans, E. H. Cho, Z. Dobbin, G. Pavlakis, and S. Lockett, “Automatic and Quantitative Measurement of Protein-Protein Colocalization in Live Cells,” *Biophys J*, vol. 86, no. 6, pp. 3993–4003, Jun. 2004, doi: 10.1529/BIOPHYSJ.103.038422.
